# Influence of entropy on Brinkman–Forchheimer model of MHD hybrid nanofluid flowing in enclosure containing rotating cylinder and undulating porous stratum

**DOI:** 10.1038/s41598-021-03477-4

**Published:** 2021-12-21

**Authors:** Fares Redouane, Wasim Jamshed, S. Suriya Uma Devi, Belhadj M. Amine, Rabia Safdar, Khaled Al-Farhany, Mohamed R. Eid, Kottakkaran Sooppy Nisar, Abdel-Haleem Abdel-Aty, I. S. Yahia

**Affiliations:** 1 LGIDD, Ahmed ZABANA University, Relizane, Algeria; 2 Department of Mathematics, Capital University of Science and Technology (CUST), Islamabad, 44000 Pakistan; 3 Department of Mathematics, KPR Institute of Engineering and Technology, Coimbatore, 641407 India; 4 Department of Mathematics, Lahore College for Women University, Lahore, 54000 Pakistan; 5 Department of Mechanical Engineering, University of Al-Qadisiyah, Al-Qadisiyah, 58001 Iraq; 6 Department of Mathematics, Faculty of Science, New Valley University, Al-Kharga, Al-Wadi Al-Gadid, 72511 Egypt; 7 Department of Mathematics, Faculty of Science, Northern Border University, Arar, 1321 Saudi Arabia; 8 Department of Mathematics, College of Arts and Sciences, Prince Sattam Bin Abdulaziz University, Wadi Aldawaser, 11991 Saudi Arabia; 9 Department of Physics, College of Sciences, University of Bisha, P.O. Box 344, Bisha, 61922 Saudi Arabia; 10 Physics Department, Faculty of Science, Al-Azhar University, Assiut, 71524 Egypt; 11 Laboratory of Nano-Smart Materials for Science and Technology (LNSMST), Department of Physics, Faculty of Science, King Khalid University, P.O. Box 9004, Abha, 61413 Saudi Arabia; 12 Research Center for Advanced Materials Science (RCAMS), King Khalid University, P.O. Box 9004, Abha, 61413 Saudi Arabia; 13 Nanoscience Laboratory for Environmental and Biomedical Applications (NLEBA), Semiconductor Lab., Department of Physics, Faculty of Education, Ain Shams University, Roxy, Cairo, 11757 Egypt

**Keywords:** Mathematics and computing, Physics

## Abstract

The current article aims to discuss the natural convection heat transfer of Ag/Al_2_O_3_-water hybrid filled in an enclosure subjected to a uniform magnetic field and provided with a rotating cylinder and an inner undulated porous layer. The various thermo-physical parameters are investigated such as Rayleigh number ($100 \le Ra \le 100000$), Hartmann number ($0 \le Ha \le 100$), and the nanoparticles concentration ($0.02 \le \phi \le 0.08$). Likewise, the rotational speed of the cylinder ($- 4000 \le \omega \le + 4000$), as well as several characteristics related to the porous layer, are examined li its porosity ($0.2 \le \varepsilon \le 0.8$), Darcy number ($- 100000 \le Da \le - 100$) which indicates the porous medium permeability and the number of undulations ($0 \le N \le 4$). The calculations are carried out based on the Galerkin Finite element method (GFEM) to present the streamlines, isotherms, entropy generation, and average Nusselt numbers in details. The main results proved that increment of Rayleigh number and Darcy number enhances heat transfer convection within the enclosure. Whilst, the porosity presents a minimal impact. Also, the rotational speed in a positive direction has a favorable influence on the heat transfer dispersion across the cavity.

## Introduction

In recent years, the natural conjugate heat transfer magnetohydrodynamics (MHD) has been an inspiring topic for researchers due to its wide use in various sectors. Such as Boilers and cooling systems, thermal energy, and several engineering applications. Additionally, the focus of the research was on the mechanism of nanofluid heat transfer^1–8^. Pordanjani et al.^9^ studied the free convection of the alumina-water nanofluid in a cavity. They examined the effect of the magnetic flux on heat transfer efficiency. Alnaqi et al.^10^ studied the magnetic field impact of the Al_2_O_3_-water nanofluid in the inclined square cavity. It was exposed from their findings, the Nusselt number increase for an upper Rayleigh number and a lower Hartmann number. In an additional study, Pordanjani et al.^11^ investigated the nanofluid heat transfer and total entropy generation $S_{gen}$ in a cavity. In the presence of magnetic flux, they retained distinct temperature profiles on the left side of the cavity divider. The upper and lower sides were insulated and the right wall was kept at low temperatures. It was found that S_gen_ was increasing for a higher $Ra$ and a lower $Ha$. Fares et al.^12^ investigated the behaviour of the free convection heat inside a porous cavity equipped with a rotating cylinder. Their findings confirmed the significant boosting impact of increasing $Ra$ and $Da$ on the heat exchange efficiency. The computational study conducted by Mebarek-Oudina et al.^13^ on the convection heat transfer inside a porous chamber filled with Ag-MgO hybrid nanofluid indicated that rising the Rayleigh number leads to accelerate the fluid flow strength. However, the increment of $Ha$ has a reducing effect. Saffarian et al.^14^ investigated the Heat transfer response in a flat plate solar collector with the presence of different shapes of flow path using nanofluid. They found that using nanofluid instead of water enhances the heat transfer intensity increases in all cases.

In addition, Selimefendigil and Öztop^15^ analyzed the properties of convection and entropy generation in the enclosure with the existence of a magnetic field. The corner was divided into two parts by a perpendicular park. The upper and base walls were adiabatic, while the left and the right walls were the hot and the cold parts. They noticed a better response of heat transfer with a greater grand a lower $Ha$. Moreover, Selimefendigil and Öztop^16^ studied the properties of the convection and $S_{gen}$ in a divided cavity filled with carbon nanotube (CNT)/water nanofluid under a magnetic flux using the finite element calculation method. The parameters were modified and the effect of the pack in the chamber was considered. Investigators found that the normal $Nu$ rises with a greater $Ra$ and a reduced $Ha$. In the study conducted by Gangawane and Bharti^17^, In a partly differently heated box, a cooler size effect was tested on MHD natural convection using the Boltzmann grid approach. It has been shown that the $Nu_{avg}$ is proportionally influenced by the cooler length and $Ra$, while it has an opposite relationship with $Ha$. In addition, Abbassi and Orfi^18^ conducted a simulation analysis using lattice-Boltzmann method (LBM) on MHD free convection of a heated block situated on the base of an enclosure filled with nanofluids. They demonstrate that the magnetic flow has the reverse effect on heat transfer efficiency, the fluid flow, and the total $S_{gen}$. The maximum heat transfer is obtained when the angle of inclination is equal to $\pi /2$. Additionally, through the study done by Esmaeil^19^, The results of thermophysical properties on natural convection of laminar in containers in which nanofluid works is measured by using the technique of finite difference. Nanoparticles are subject to Brownian movement and thermophoresis physical transport processes. It is found that the key parameter that influences the flow of heat from nanofluids is the nanofluid viscosity. A theoretical analysis was carried out in a porous cavity filled with Cu/water nanofluid with LBM by Hoseinpour et al.^20^. In this analysis, the total $S_{gen}$ is found to be decreased and the $Nu_{avg}$ increases as the nanofluid volume fraction is raised. Also, the total $S_{gen}$. is strongly affected by porous porosity. It is found that as the porosity is greater, the total $S_{gen}$ improves. Abu-Libdeh et al.^21^ studied the impact of the deferent thermos-physical parameters on the hydrothermal and the entropy generation inside a novel porous cavity. Kasaeipoor et al.^22^ conducted a free convection heat transfer and entropy generation analysis in an enclosure with refrigerant solid elements loaded with MWCNT–MgO/water hybrid nanofluid. It was inferred that the larger refrigerant solid body improves the $Nu_{avg}$ and $S_{gen}$. In comparison, the $Nu_{avg}$ greatly increases improving $\phi$ and then decreases. With the rise of $Ra$, the $S_{gen}$ is enhanced and decreases with $\phi$. Rahimi et al.^23^ performed a numerical study investigating natural convection and entropy generation within an enclosure. Partially active walls, charged with two walls of carbon nanotubes, nanofluid water and fitted with cold, and hot barriers are provided, while the device is subject to LBM. The obtained results revealed that the $Nu_{avg}$ improves when $Ra$ and $\phi$ increase. However, the $S_{gen}$ increase with $Ra$ and decreases when $\phi$ rises up. additionally, Rahimi et al.^24^ normal convection heat transfer and water-CuO nanofluid entropy generation in a square chamber supplied with fins. They noticed that by improving $Ra$ and $\phi$, the $Nu_{avg}$ enhances. Also, they found that $S_{gen}$ reduced by the increasing of $\phi$. Fares et al.^25^ discussed the optimization of the entropy generation inside a square cavity loaded with Ag/water nanofluid. Mainly, they investigated the effect of inclined magnetic field on $S_{gen}$ and $Nu_{avg}$ responses. Alsabery et al.^26^ investigated the Impact of the use of two-phase hybrid nanofluid on mixed convection within a wavy lid-driven enclosure and equipped with a solid block. They found that the position of the solid block and surface undulation are significant in controlling heat transmission and the concentration distribution of the composite nanoparticles. Tayebi and Chamkha^27^ studied MHD heat transfer within a nanofluid filled-square chamber separated by a solid conductive wall. Their findings showed that the combined impacts of the varied vertical conducting wall designs and other relevant factors can be an efficient way of regulating flow characteristics and heat transfer rate inside the system. Mebarek-Oudina et al.^28^ studied the heat transfer and the entropy generation in case of magnetized hybrid nanoliquid flow involved in a trapezoidal enclosure. They demonstrated that increasing the Rayleigh number and reducing the Hartmann number enhances the thermal efficiency of the chamber. Belhadj et al.^29^ investigated the nanofluid natural convection response inside a triangular cavity with an inner partial porous media installed at the right-angled corner. They noticed that the increase in Darcy number and the porosity has a boosting effect on the heat transfer efficiency. This infuence is more intensified whith greater Rayleigh and lower Hartmann numbers. Alsabery et al.^30^ studied the entropy generation and mixed Convection heat transfer inside a cavity equipped with wavy walls and rotating Solid Cylinder. They found that the flow can be controlled by adjusting the cylinder's angular velocity. Moreover, they noticed that the clockwise rotation around the solid cylinder intensifies the convective flow cell inside the wavy container as the Rayleigh number rises. At the top portion of the heated surface, the local Nusselt number peaks. Brahimi et al.^31^ conducted a numerical study of thermal and streamline analysis inside a cavity filled with (Ag–MgO/Water) hybrid nanofluid. They determined that because the box's structure causes the flow to meander over the cliff bars, this unique flux tends to slow the flow around it, allowing the particles to thermally transfer.

The LBM and modified LBM in an enclosure has been studied by^32–34^ in conjugate natural convection and diffusion. They found that the heat transfer and the total $S_{gen}$ increase when $Ra$ decreased the number of Bejan. It has been observed that as the thermal conductivity ratio rises, the $Nu$ and the total $S_{gen}$ rate rise, however, be reduced. In addition, it is important to note that several recent researches have been performed to examine rarefied flow activity and heat transfer response along with $S_{gen}$ within enclosures^35,36^. It is noted that Knudsen number improves, the transfer efficiency reduces. Furthermore, LBM has later been used as an integrated approach focusing on kinetic theory and as a novel solution to discretization approaches^37^. Additionally, LBM presents second-order precision in both time and space; it is simple to code and can be used to manage a wide variety of flow patterns varying from microscopic to continuous levels.

As shown in the relevant literature above, convection heat transfer and entropy generation in such complex geometry subjected to a uniform magnetic field with the presence of an inner corrugated porous layer has never been studied previously. Indeed, the purpose of the current research is to analyze numerically the MHD convective heat transfer and the entropy generation for a partially differentially heated divided enclosure filled by Ag/Al_2_O_3_-water. The computational accuracy is based on LBM for a two-dimensional approximation of the governing equations. Experimental correlations for thermal and physical characteristics of nanofluids are used in this study like thermal conductivity and dynamic viscosity. The heat transfer and $S_{gen}$ within the partitioned cavity is investigated using dimensionless independent parameters such as $Ra$, nanoparticles fraction volume, $Ha$, and the magnetic field tilting angle. Furthermore, the heat generating rod bundle for nuclear applications, which may be considered as a heat generating anisotropic porous medium, is an example of particular real-world uses of the current situation.

## Problem description

**Figure 1:**
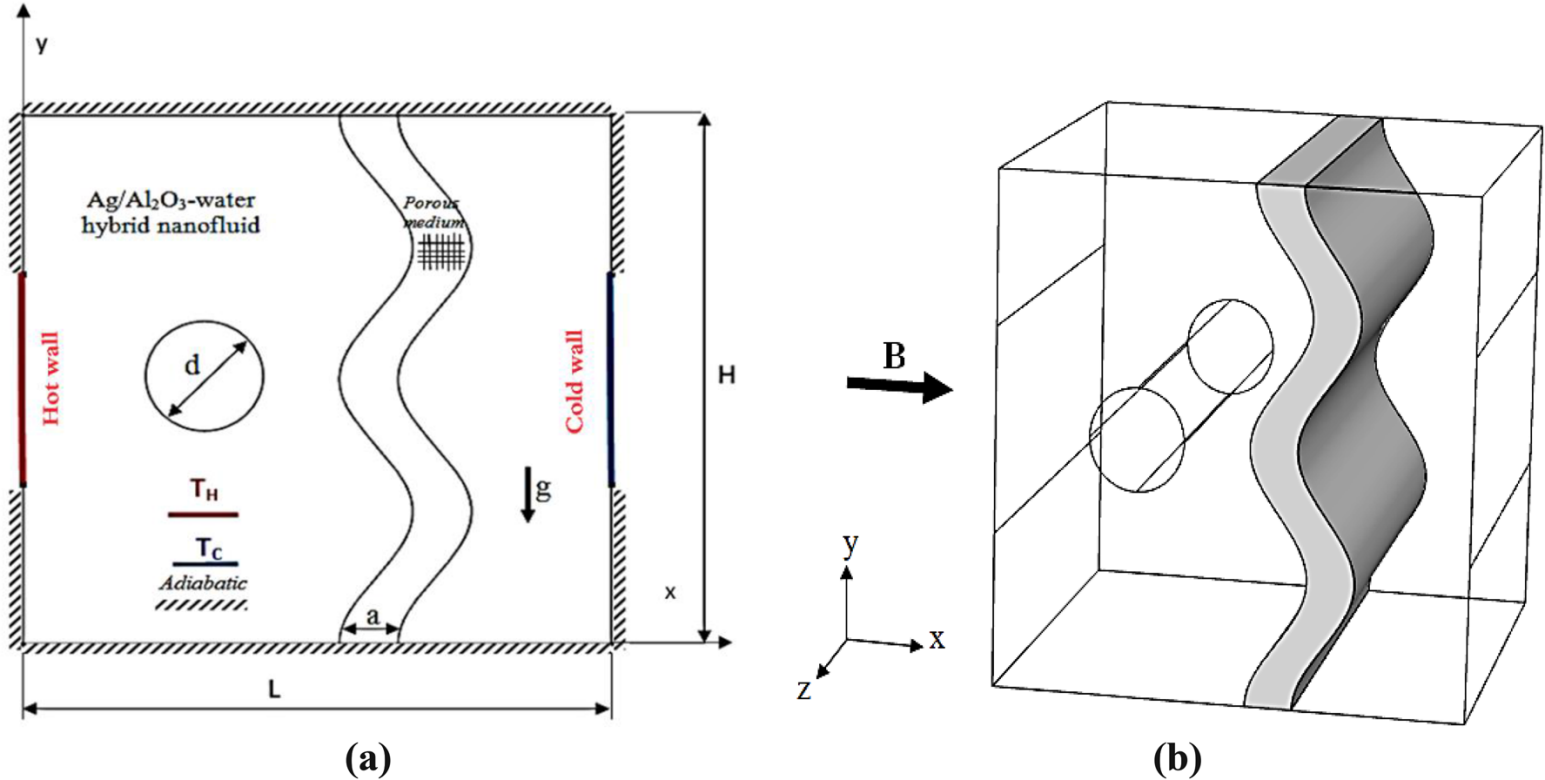
Schematic diagram of the physical model in (**a**) 2D and (**b**) 3D.

The geometry studied is seen in Fig. 1. Representation shown in 2D (a) and 3D (b). The top and the bottom walls are insulated. In addition, both sidewalls are equipped with hot and cold parts while the temperatures are upheld as $T_{h}$ and $T_{c}$ on the left and right walls respectively. The heater and cooler parts have been used as ($0.4H$) for all cases and it is symmetrical on the center of the vertical walls. Whereas, all remaining walls are insulated. An undulated vertical porous layer with a thickness ($a = 0.1L$) has been placed in the cavity at ($b = 0.35L$). A rotating circular cylinder with a diameter ($d_{m} = 0.2L$) is placed in the middle of the cavity at a distance of ($c = 0.3L$) on the $x -$ axis. A uniform magnetic field is applied to the cavity in $x -$ direction. The cavity is filled with the (Ag/Al_2_O_3_-water) hybrid nanofluid, which are considered Newtonian, incompressible with no viscous dissipation, and laminar flow. The thermophysical properties of hybrid nanofluid Ag/Al_2_O_3_-water are listed in Table 1. The water Prandtl number is specified to be $Pr = 6.2$.

**Table 1:** Thermophysical properties of the base fluid and nanoparticles Ag/Al_2_O_3_ (50%/50%)^12,38^.

Material	$C_{p} \left( {{\text{J/kg}}\,{\text{K}}} \right)$	${\text{k}}\left( {{\text{W/m}}\,{\text{k}}} \right)$	$\rho \left( {{\text{kg}}/{\text{m}}^{3} } \right)$	$\beta \times 10^{ - 5} \,\,\left( {{\text{K}}^{ - 1} } \right)$	$\sigma \left( {\text{s/m}} \right)$	$\alpha \,\,\left( {{\text{m}}^{2} /{\text{s}}} \right)$
Water	4179	0.613	997.1	21	5.5 × 10^–6^	1.47 × 10^–7^
Ag	235	429	10,500	5.4	8.1 × 10^–4^	147 × 10^–3^
Al_2_O_3_	765	40	3970	0.85	2.7 × 10^–8^	131.79 × 10^–7^

The principal thermophysical characteristics of the base fluid (water) and the added nanoparticles are given in the following Table 1.

## Formulation of mathematical model

### Governing equations and boundary conditions:

Via a novel shape of a porous cavity for hybrid nanofluid, the stationary natural MHD convective flow is investigated. The Darcy-Brinkman-Forchheimer model^39^ is being used for the numerical modelling of the porous media. To be formulated in a dimension model, Navier–Stokes and heat equations, expressed in Cartesian coordinates for the present study with the above assumptions in mind, can be given as follows:

In 2D cavity fluid domain, the main conservation equations in the hybrid-nanofluid region are the followings^40^:

1 $$ \frac{\partial u}{{\partial x}} + \frac{\partial v}{{\partial y}} = 0, $$

2 $$ u\frac{\partial u}{{\partial x}} + v\frac{\partial u}{{\partial y}} = - \frac{1}{{\rho_{hnf} }}\frac{\partial P}{{\partial x}} + \nu_{hnf} \left( {\frac{{\partial^{2} u}}{{\partial x^{2} }} + \frac{{\partial^{2} u}}{{\partial y^{2} }}} \right), $$

3 $$ u\frac{\partial v}{{\partial x}} + v\frac{\partial v}{{\partial y}} = - \frac{1}{{\rho_{hnf} }}\frac{\partial P}{{\partial y}} + \nu_{hnf} \left( {\frac{{\partial^{2} v}}{{\partial x^{2} }} + \frac{{\partial^{2} v}}{{\partial y^{2} }}} \right) + \beta_{hnf} g\left( {T - T_{avg} } \right) - \frac{{\sigma_{hnf} }}{{\rho_{hnf} }}B_{0}^{2} v, $$

4 $$ (\rho c_{p} )_{hnf} \left( {u\frac{\partial T}{{\partial x}} + v\frac{\partial T}{{\partial y}}} \right) = k_{hnf} \left( {\frac{{\partial^{2} T}}{{\partial x^{2} }} + \frac{{\partial^{2} T}}{{\partial y^{2} }}} \right). $$

According to Darcy–Brinkmann–Forchheimer generalized equation, the governing equations for the porous region can be written as^12^:

5 $$ \frac{\partial u}{{\partial x}} + \frac{\partial v}{{\partial y}} = 0, $$

6 $$ \frac{1}{{\varepsilon^{2} }}\left( {u\frac{\partial u}{{\partial x}} + v\frac{\partial u}{{\partial y}}} \right) = - \frac{1}{{\rho_{hnf} }}\frac{\partial P}{{\partial x}} + \frac{{\nu_{hnf} }}{\varepsilon }\left( {\frac{{\partial^{2} u}}{{\partial x^{2} }} + \frac{{\partial^{2} u}}{{\partial y^{2} }}} \right) - \nu_{hnf} \frac{u}{K} - \frac{{F_{c} }}{\sqrt K }u\left| u \right|, $$

7 $$ \frac{1}{{\varepsilon^{2} }}\left( {u\frac{\partial v}{{\partial x}} + v\frac{\partial v}{{\partial y}}} \right) = - \frac{1}{{\rho_{hnf} }}\frac{\partial P}{{\partial y}} + \frac{{\nu_{hnf} }}{\varepsilon }\left( {\frac{{\partial^{2} v}}{{\partial x^{2} }} + \frac{{\partial^{2} v}}{{\partial y^{2} }}} \right) - \nu_{hnf} \frac{v}{K} - \frac{{F_{c} }}{\sqrt K }v\left| u \right| + \beta_{hnf} g\left( {T - T_{avg} } \right) - \frac{{\sigma_{hnf} }}{{\rho_{hnf} }}B_{0}^{2} v, $$

8 $$ (\rho c_{p} )_{hnf} \left( {u\frac{\partial T}{{\partial x}} + v\frac{\partial T}{{\partial y}}} \right) = k_{hnf} \left( {\frac{{\partial^{2} T}}{{\partial x^{2} }} + \frac{{\partial^{2} T}}{{\partial y^{2} }}} \right), $$

where, $\left| u \right| = \sqrt {u^{2} + v^{2} }$. Forchheimer coefficient $F_{c} = \frac{b}{{\sqrt a \varepsilon^{3/2} }}$ (where $a = 150$ and $b = 1.75$) represents the operative thermal conductivity of porous media saturated with nanofluid, where $K$ is the porous medium permeability and $\varepsilon$ is its porosity, described as follows^38,41^:

9 $$ K = \frac{{\varepsilon^{3} d_{m}^{2} }}{{150\left( {1 - \varepsilon } \right)^{2} }} $$

To reformulate the previous governing equations into non-dimensional ones, the following variables are used:

10 $$ X = \frac{x}{L},Y = \frac{y}{L},U = \frac{uL}{{\alpha_{bf} }},V = \frac{vL}{{\alpha_{bf} }}, \theta = \frac{{T - T_{f} }}{{T_{h} - T_{f} }},P = \frac{{\left( {p + \rho_{bf} g_{y} } \right)L^{2} }}{{\rho_{bf} \alpha_{bf}^{2} }}. $$

Dimensionless numbers are given as follow:

11 $$ Ra = \frac{{\beta_{bf} g\left( {T_{h} - T_{f} } \right)L^{3} }}{{\alpha_{bf} v_{bf} }},Ha = LB_{0} \sqrt {\frac{{\sigma_{bf} }}{{\mu_{bf} }}} , Da = \frac{K}{{L^{2} }},Pr = \frac{{v_{bf} }}{{\alpha_{bf} }}, $$

The non-dimensional equations in the hybrid-nanofluid region can be written as:

12 $$ \frac{\partial U}{{\partial X}} + \frac{\partial V}{{\partial Y}} = 0, $$

13 $$ U\frac{\partial U}{{\partial X}} + V\frac{\partial U}{{\partial Y}} = - \frac{\partial P}{{\partial X}} + \Pr \frac{{\rho_{f} }}{{\rho_{hnf} }}\frac{{\mu_{hnf} }}{{\mu_{f} }}\left( {\frac{{\partial^{2} U}}{{\partial X^{2} }} + \frac{{\partial^{2} U}}{{\partial Y^{2} }}} \right), $$

14 $$ U\frac{\partial V}{{\partial X}} + V\frac{\partial V}{{\partial Y}} = - \frac{\partial P}{{\partial Y}} + \Pr \frac{{\rho_{f} }}{{\rho_{hnf} }}\frac{{\mu_{hnf} }}{{\mu_{f} }}\left( {\frac{{\partial^{2} V}}{{\partial X^{2} }} + \frac{{\partial^{2} V}}{{\partial Y^{2} }}} \right) + Ra Pr\frac{{\rho_{f} }}{{\rho_{hnf} }}\left[ {1 - \phi + \phi \frac{{\left( {\rho \beta } \right)_{hnf} }}{{\left( {\rho \beta } \right)_{f} }}} \right]\theta , $$

15 $$ U\frac{\partial \theta }{{\partial X}} + V\frac{\partial \theta }{{\partial Y}} = \frac{{\alpha_{hnf} }}{{\alpha_{bf} }}\left( {\frac{{\partial^{2} \theta }}{{\partial X^{2} }} + \frac{{\partial^{2} \theta }}{{\partial Y^{2} }}} \right). $$

The non-dimensional equations in the porous region can be written as^42^:

16 $$ \frac{\partial U}{{\partial X}} + \frac{\partial V}{{\partial Y}} = 0, $$

17 $$ \frac{1}{{\varepsilon^{2} }}\frac{{\rho_{hnf} }}{{\rho_{bf} }}\left( {U\frac{\partial U}{{\partial X}} + V\frac{\partial U}{{\partial Y}}} \right) = - \frac{\partial P}{{\partial X}} + \frac{1}{\varepsilon }\frac{{\nu_{hnf} }}{{\nu_{bf} }}\Pr \left( {\frac{{\partial^{2} U}}{{\partial X^{2} }} + \frac{{\partial^{2} U}}{{\partial Y^{2} }}} \right) - \frac{{\nu_{hnf} }}{{\nu_{bf} }}\frac{Pr}{{Da}} - \frac{{F_{c} }}{{\sqrt {Da} }}\left| u \right|U, $$

18 $$ \frac{1}{{\varepsilon^{2} }}\frac{{\rho_{hnf} }}{{\rho_{f} }}\left( {U\frac{\partial V}{{\partial X}} + V\frac{\partial V}{{\partial Y}}} \right) = - \frac{\partial P}{{\partial Y}} + \frac{1}{\varepsilon }\frac{{\nu_{hnf} }}{{\nu_{f} }}Pr\left( {\frac{{\partial^{2} V}}{{\partial X^{2} }} + \frac{{\partial^{2} V}}{{\partial Y^{2} }}} \right) - \frac{{\nu_{hnf} }}{{\nu_{f} }}\frac{Pr}{{Da}}V - \frac{Fc}{{\sqrt {Da} }}\left| u \right|V + \frac{{\beta_{hnf} }}{{\beta_{f} }}Pr \cdot Ra\theta + \frac{{\sigma_{f} }}{{\rho_{hnf} }}\frac{{\rho_{f} }}{{\rho_{hnf} }}\frac{{\Pr Ha^{2} }}{{\varepsilon \sqrt {Ra} }}V, $$

19 $$ U\frac{\partial \theta }{{\partial X}} + V\frac{\partial \theta }{{\partial Y}} = \frac{{\alpha_{hnf} }}{{\alpha_{bf} }}\left( {\frac{{\partial^{2} \theta }}{{\partial X^{2} }} + \frac{{\partial^{2} \theta }}{{\partial Y^{2} }}} \right). $$

The relationships between the velocity and the stream function are^43^:

20 $$ \left\{ {\begin{array}{*{20}c} {U = \frac{\partial \psi }{{\partial Y}}, } \\ {V = - \frac{\partial \psi }{{\partial X}},} \\ \end{array} } \right. $$

a single equation become,

21 $$ \frac{{\partial^{2} \psi }}{{\partial X^{2} }} + \frac{{\partial^{2} \psi }}{{\partial y^{2} }} = \frac{\partial U}{{\partial Y}} - \frac{\partial V}{{\partial X}} $$

### Non-dimensional Entropy Generation

Local entropy in hybrid nanofluid region production measurement was obtained from totaling the conjugated fluxes and the forces developed. The non-dimensional local entropy production is given by Woods^44^ in a convective process:

22 $$ S_{gen} = \frac{{k_{hnf} }}{{k_{bf} }}\left[ {\left( {\frac{\partial \theta }{{\partial X}}} \right)^{2} + \left( {\frac{\partial \theta }{{\partial Y}}} \right)^{2} } \right] + \chi \frac{{\mu_{hnf} }}{{\mu_{bf} }}\left\{ {\left( {U^{2} + V^{2} } \right) + Da\left[ {2\left( {\frac{\partial U}{{\partial X}}} \right)^{2} + 2\left( {\frac{\partial V}{{\partial Y}}} \right)^{2} + \left( {\frac{\partial U}{{\partial Y}} + \frac{\partial V}{{\partial X}}} \right)^{2} } \right]} \right\} + \frac{{\sigma_{hnf} }}{{\sigma_{f} }}\chi Ha^{2} V^{2} $$

23 $$ \chi = \frac{{\mu_{hnf} T_{avg} }}{{k_{bf} K}}\left( {\frac{{\alpha_{bf} }}{{L\left( {T_{H} - T_{C} } \right)}}} \right)^{2} ,T_{avg} = \frac{{T_{H} + T_{C} }}{2} $$

For boundary conditions related to the walls of the studied cavity, the dimensional former presented as following:

- The hot wall:
  24 $$ u = v = 0, T = T_{h} . $$
- The cold Wall:
  25 $$ u = v = 0, T = T_{c} . $$
- The insolated walls:
  26 $$ u = v = 0, \frac{\partial T}{{\partial n}} = 0. $$
- Over the rotating cylinder
  27 $$ \left\{ {\begin{array}{*{20}c} {u = - \omega \left( {y - y_{0} } \right)} \\ {v = - \omega \left( {x - x_{0} } \right)} \\ \end{array} } \right. $$
  28 $$ Nu_{loc} = - \frac{{k_{hnf} }}{{k_{bf} }}\frac{\partial \theta }{{\partial X}}, $$
  29 $$ Nu_{avg} = \mathop \smallint \limits_{0}^{L} Nu_{loc} dY. $$

### Thermophysical characteristics of the hybrid-nanofluid

The density and the thermal conductivity as well as the heat capacity of the hybrid nanofluid can be given as the following^29,31^

30 $$ \rho_{hnf} = \left[ {\left( {1 - \phi_{2} } \right)\left\{ {\left( {1 - \phi_{1} } \right)\rho_{f} + \phi_{1} \rho_{{p_{1} }} } \right\}} \right] + \phi_{2} \rho_{{p_{2} }} $$

31 $$ \sigma_{hnf} = \left[ {\left( {1 - \phi_{2} } \right)\left\{ {\left( {1 - \phi_{1} } \right)\sigma_{bf} + \phi_{1} \sigma_{p1} } \right\}} \right] + \phi_{2} \sigma_{p2} , $$

32 $$ {(\rho \beta_{p} )}_{hnf} = [( {1 - \phi_{2} } )\{ ( {1 - \phi_{1} } )( {\rho \beta_{p} )_{f} + \phi_{1} {(}\rho \beta_{p} )_{{p_{1} }} \}} ] + \phi_{2} (\rho \beta_{p} )_{{p_{2} }} , $$

33 $$ (\rho C_{p} )_{hnf} = [\left( {1 - \phi_{2} } \right)\{ \left( {1 - \phi_{1} } \right)( {\rho C_{p} )_{f} + \phi_{1} {(}\rho C_{p} )_{{p_{1} }} \} } ] + \phi_{2} (\rho C_{p} )_{{p_{2} }} , $$

34 $$ \alpha_{hnf} = \frac{{k_{hnf} }}{{\left( {\rho c_{p} } \right)_{hnf} }}, $$

35 $$ \frac{{\kappa_{hnf} }}{{\kappa_{f} }} = \left[ {\frac{{\left( {\kappa_{{p_{2} }} + 2\kappa_{bf} } \right) - 2\phi_{2} \left( {\kappa_{bf} - \kappa_{{p_{2} }} } \right)}}{{\left( {\kappa_{{p_{2} }} + 2\kappa_{bf} } \right) + \phi_{2} \left( {\kappa_{bf} - \kappa_{{p_{2} }} } \right)}}} \right]* \left[ {\frac{{\left( {\kappa_{{p_{1} }} + 2\kappa_{f} } \right) + \phi_{1} \left( {\kappa_{f} - \kappa_{{p_{1} }} } \right)}}{{\left( {\kappa_{{p_{1} }} + 2\kappa_{f} } \right) - 2\phi_{1} \left( {\kappa_{f} - \kappa_{{p_{1} }} } \right)}}} \right], $$

The effective dynamic viscosity of the hybrid nanofluid based on the Brinkman mode is considered as^29,31^:

36 $$ \mu_{hnf} = \mu_{f} (1 - \phi_{1} )^{ - 2.5} (1 - \phi_{2} )^{ - 2.5} $$

37 $$ \frac{{\sigma_{hnf} }}{{\sigma_{f} }} = \left[ {\frac{{\left( {\sigma_{{p_{2} }} + 2\sigma_{bf} } \right) - 2\phi_{2} \left( {\sigma_{bf} - \sigma_{{p_{2} }} } \right)}}{{\left( {\sigma_{{p_{2} }} + 2\sigma_{bf} } \right) + \phi_{2} \left( {\sigma_{bf} - \sigma_{{p_{2} }} } \right)}}} \right]* \left[ {\frac{{\left( {\sigma_{{p_{1} }} + 2\sigma_{f} } \right) + \phi_{1} \left( {\sigma_{f} - \sigma_{{p_{1} }} } \right)}}{{\left( {\sigma_{{p_{1} }} + 2\sigma_{f} } \right) - 2\phi_{1} \left( {\sigma_{f} - \sigma_{{p_{1} }} } \right)}}} \right], $$

where, $\phi$ signifies the nanoparticle concentration factor. $\mu_{f}$, $\rho_{f}$, $(C_{p} )_{f}$ and $\kappa_{f}$ are fluid viscidness, consistency, operative heat capacitance and thermally conductance of the basefluid, correspondingly. $\mu_{hnf}$, $\rho_{hnf}$, $\rho (C_{p} )_{hnf}$ and $\kappa_{hnf}$ are hybrid nanofluid dynamical viscidness, intensity, specific heat capacitance and thermal conductance. $\mu_{f}$, $\rho_{f}$, $(C_{p} )_{f}$, $\kappa_{f}$ and $\sigma_{f}$ are dynamical viscidness, density, specific heat capacitance and thermal conductance of the basefluid. $\rho_{{p_{1} }}$, $\rho_{{p_{2} }}$, $(C_{p} )_{{p_{1} }}$, $(C_{p} )_{{p_{2} }}$, $\kappa_{{p_{1} }}$ and $\kappa_{{p_{2} }}$ are the intensity, specific heat capacitance and thermal conductance of the nanomolecules.

## Validation and grid independence analysis

**Figure 2:**
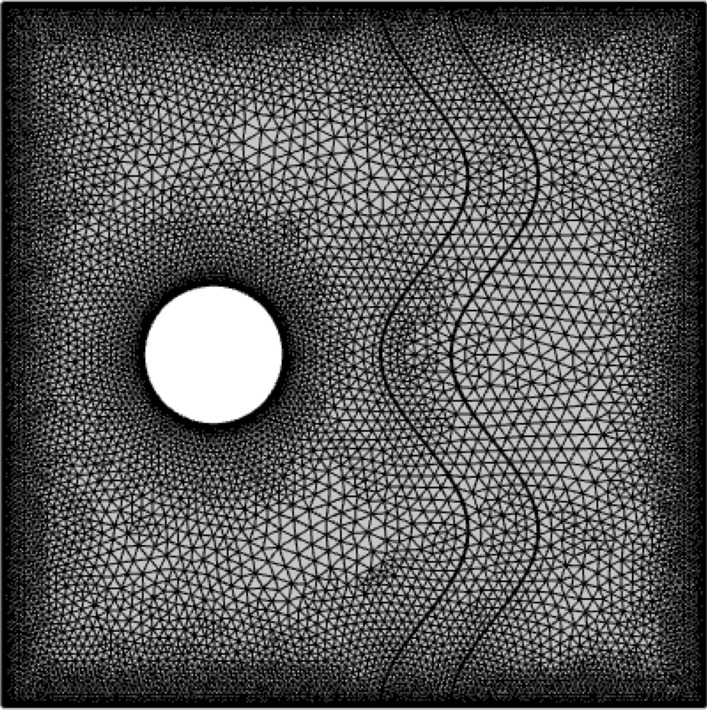
Sample mesh representation of the problem.

**Figure 3:**
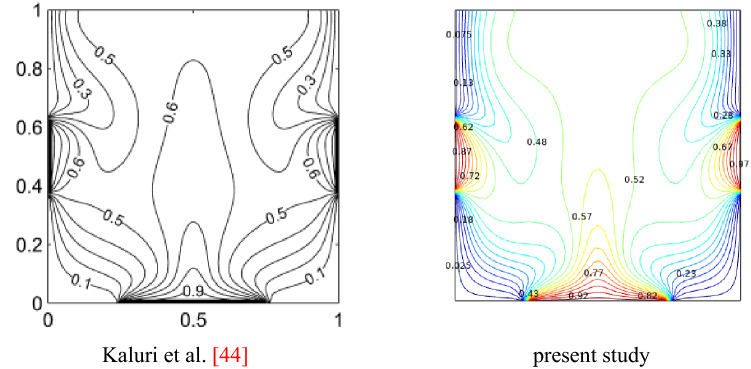
Numerical results from Kaluri et al.^45^ versus the present study for streamlines at $Ra = 10^{6}$.

Seven different grids were used to confirm that the results were not dependent on the grid. The independence of flow and heat transfer from the number of grids is determined using $Nu_{avg}$, the stream function, and general entropy (see Table 2). Due to the different results, the sixth grid was preferred as the final grid for all cases as shown in Fig. 2. Ensuring the numerical solution method is one of the primary criteria for achieving results, previous studies of Kaluri et al.^45^ were used to validate our model, as shown in Fig. 3.

**Table 2:** Evaluation of the $Nu_{avg}$,$\psi_{max}$ and $S_{gen}$ for diverse grid resolution.

Mesh size	860	1520	2118	3108	7348	20,332	27,046
$Nu_{avg}$	6.6227	6.7137	6.7820	6.8316	6.9789	7.0888	7.0891
$\psi_{max}$	0.2055	0.1491	0.1509	0.1513	0.1508	0.1506	0.1503
$S_{gen}$	47.960	50.527	52.445	54.052	59.622	64.881	64.886

It is important to remember that the above governing equations along with the limits are solved by Galerkin finite element approach. Galerkin weighted residual finite element formula solves the equations numerically (We used the COMSOL Multiphysics® software to perform the modeling^46^). In triangular elements, the code environment is separated. On any of the flow variables inside the code domain, triangular Lagrange finite elements are used from various orders. Residue is generated by replacing the approximations with the governing equations for each conservation equation. A Newton–Raphson iteration algorithm has been used to simplify nonlinear terms in the momentum equations. The solution convergence is considered if the relative error for each of the variables reaches the following convergence parameters:

$$ \left| {\frac{{ \Gamma ^{i + 1} - \Gamma ^{i} }}{{\Gamma^{ i} }}} \right| \le \eta , $$

where it represents the iteration number and $\eta$ is the convergence criterion. In this study, the convergence criterion was set at $\eta = 10^{ - 6}$.

## Results and discussion

The main motivation of this article focused on, by utilizing the efficient finite element method (FEM), the crucial thermophysical properties of Ag/Al_2_O_3_-Water hybrid nanofluid inside the enclosure under the influence of consistent magnetic field over the rotating cylinder with undulated porous layer. Many parameters have been used in this study such as Rayleigh number ($Ra = 10^{2} , 10^{3} ,10^{4} ,10^{5}$), Hartmann number ($Ha = 0, 25, 50, 100$), nanoparticles concentration ($\phi = 0.02, 0.04, 0.06, 0.08$), rotational speed of the cylinder ($\omega = - 4000, - 2000, 0 , 2000, + 4000$), porosity ($\varepsilon = 0.2, 0.4, 0.6, 0.8$), Darcy number ($Da = 10^{ - 5}$, $10^{ - 4}$, $10^{ - 3}$, $10^{ - 2}$) and undulations ($N = 0, 1, 2, 4$). The results were obtained and represented graphically through streamlines and isotherms. The discussion was made on effects forge by the individual parameter to get a deep insight into this analysis.

### Effect of Rayleigh number

**Figure 4:**
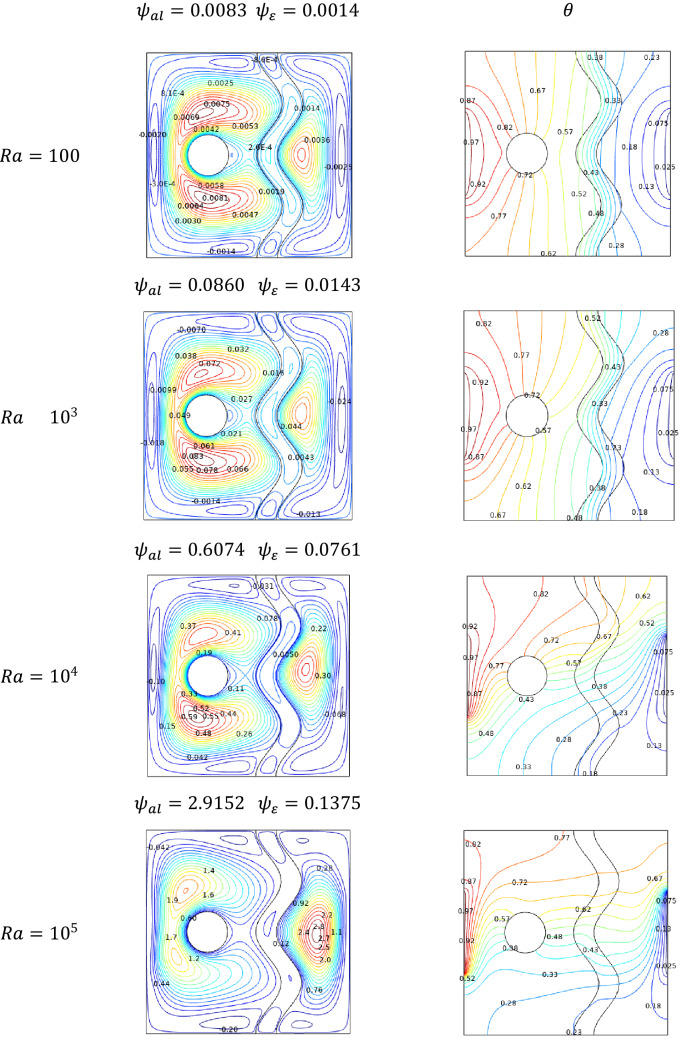
Variations of the streamlines (left), isotherms (right) with various Rayleigh number ($Ra$) at, $Ha = 0$, $N = 2$, $Da = 0.01$, $\phi = 0.02$, $\varepsilon = 0.2$ and $\omega = 0$.

Figure 4 shows the streamlines and isotherms inside the studied cavity filled with Ag/Al_2_O_3_-water nanofluid for varying Rayleigh numbers. It is noted that the streamlines formed two contours near the heated vertical wall and a single contour beside the cooled vertical wall of the cavity. For lower values of the Rayleigh number, the heated streamline contours dominate the cooled single contour. Increasing Rayleigh number ($Ra$) shifts the domination towards the cooling end. This may be due to the convectional transport across the cavity.

Isotherms for smaller $Ra$ were distributed vertically from both ends of thermal spots. For increasing values of $Ra$, isotherms were pulled towards each other. Interestingly, the heated isotherms extend up to the cooled wall occupying the top of the cavity, whereas the cooled isotherms creep through the bottom. This shows the density reduction due to heat in the cavity fluid.

### Effect of Darcy number

Effect of Darcy number becomes significant around the porous structure of the cavity. As Darcy's number increases, the permeability of the medium is increased to allow the flow into it. It was visualized by the streamline accumulation on either side of a porous medium and flows into that for higher values of Darcy number $Da$. Compare to the hotter side, the cooler side possesses intense contour, this may be due to the slower permeability of cooled fluid.

**Figure 5:**
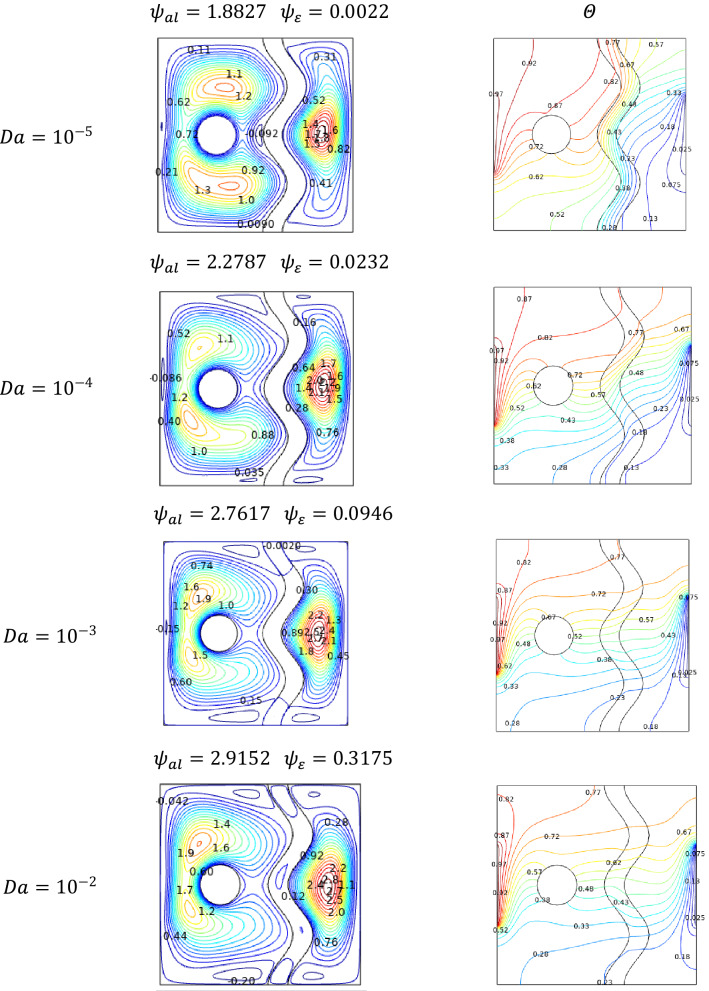
Variations of the streamlines (left), isotherms (right) with various Darcy number ($Da$) at, $Ra = 10^{5}$, $Ha = 0$, $N = 2$, $\phi = 0.02$, $\varepsilon = 0.2$ and $\omega = 0$.

The obtained results in Fig. 5 depict that as more fluid enters through the porous medium for increasing values of Darcy number $Da$, temperature around the porous medium and middle part of the cavity exhibits smoother isotherms for higher values of Darcy number $Da$.

**Figure 6:**
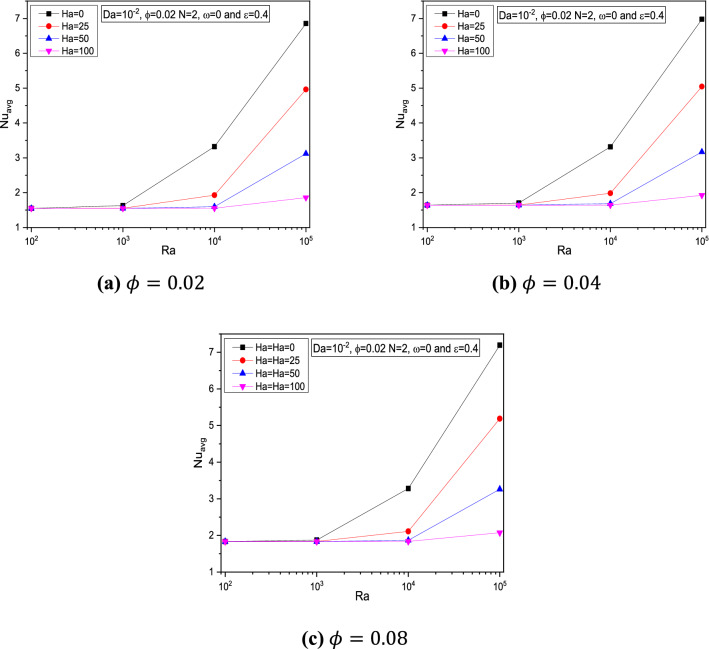
Variations of $Nu_{avg}$ with $Ra$ for various $Ha$,$\varepsilon = 0.4$, $\omega = 0, \,\,Da = 10^{ - 2}$ at (**a**) $\phi = 0.02$ (**b**) $\phi = 0.04$ and (**c**) $\phi = 0.08$.

**Figure 7:**
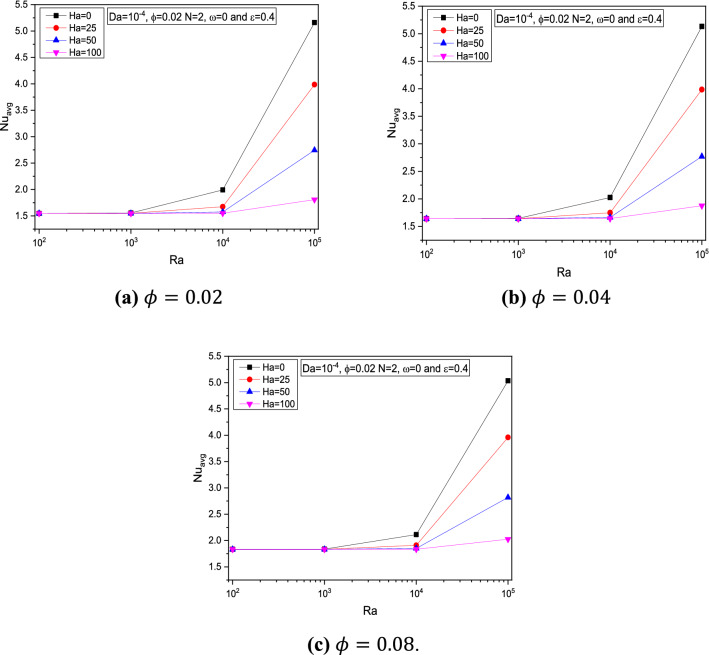
Variations of $Nu_{avg}$ with $Ra$ for various $Ha$,$\varepsilon = 0.4$, $\omega = 0$, $Da = 10^{ - 4}$, $N = 2$ at (**a**) $\phi = 0.02$ (**b**) $\phi = 0.04$ and (**c**) $\phi = 0.08$.

The state of average Nusselt number $Nu_{avg}$ across the cavity for various physical parameters were portrayed in Figs. 6 and 7. When the Rayleigh number $Ra$ increased, the heat transfer rate also gets elevated especially after $10^{2}$ for all physical parameters involved in the problem. It is found that those physical parameters like Porosity $\varepsilon$, Solid Volume Fraction $\phi$, and Darcy number $Da$ were to boosts the heat transfer rates evident through increasing average Nusselt number $Nu_{avg}$ while the Hartmann number $Ha$ tends to reduce.

### Effect of Hartmann number

**Figure 8:**
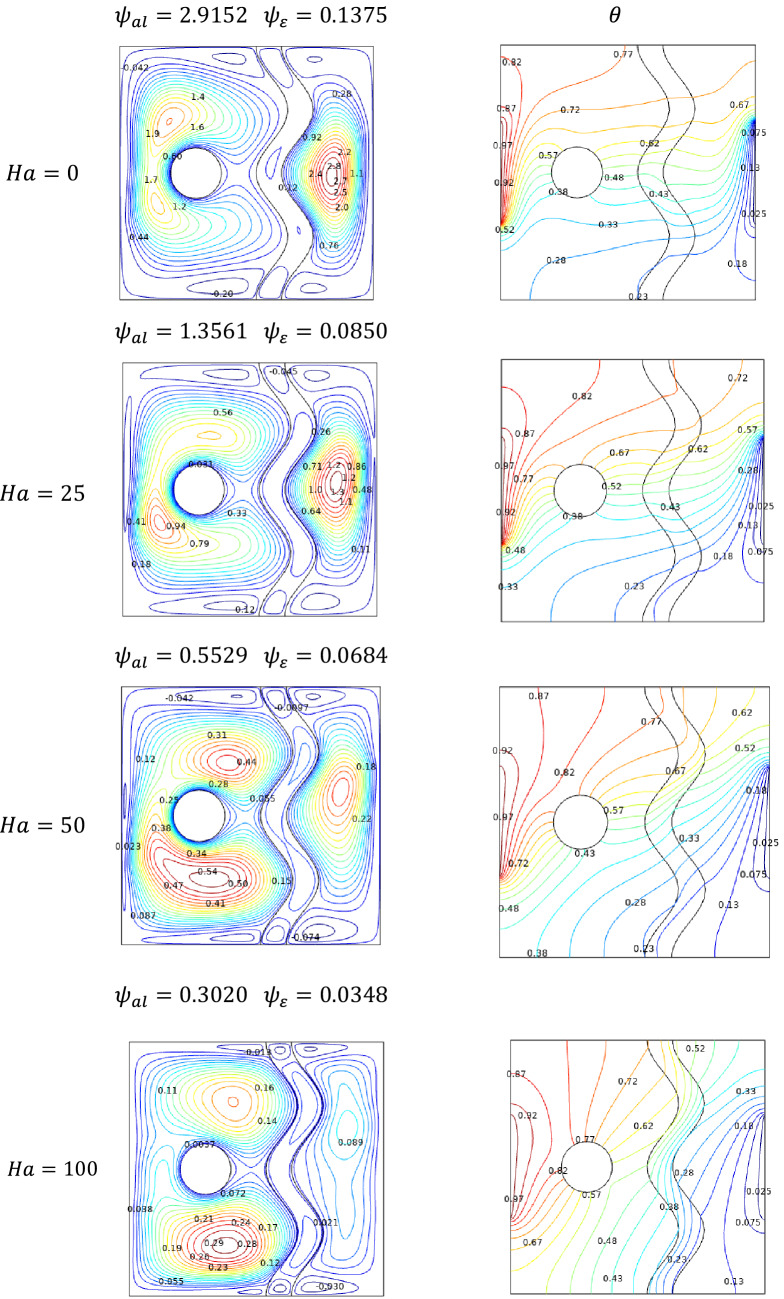
Variations of the streamlines (left), isotherms (right) with various Hartmann number ($Ha$) at $Ra = 10^{5}$, $Da = 0.01$, $N = 2$, $\phi = 0.02$, $\varepsilon = 0.2$ and $\omega = 0$.

Encountering forces induced by electromagnetic with the viscous changes by temperature variations can be modeled through Hartmann number $Ha$. Figure 8 illustrates the streamlines, isotherms inside the studied cavity with temperature differences on either side that were subjected to the transverse magnetic field $B$. In absence of magnetic field influence ($Ha = 0$), Streamlines formed a single contour near the cooler side along with the two minor contours in the heated side. Due to the increasing resistance to the flow, for higher Hartmann numbers the intensity of those contours got shifted more towards the bottom of the cavity.

Magnetically restricted stream of the fluid inside the cavity assists the slower dissipation of temperature from both ends towards each other. For higher values of $Ha$, the isotherms start to spread away from the surface. As the heated isotherms claim up the cooler isotherms cover the lower cavity regions.

**Figure 9:**
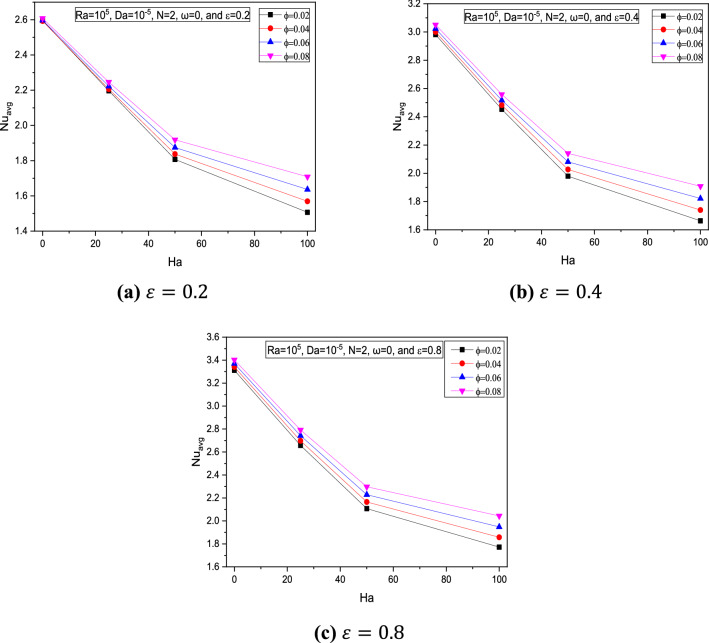
Variations of $Nu_{avg}$ with $Ha$ for various $\phi$ for $Ra = 10^{5}$, $Da = 10^{ - 2}$, and $\omega = 0$ at (**a**) $\varepsilon = 0.2$, (**b**) $\varepsilon = 0.4$, and (**c**) $\varepsilon = 0.8$.

**Figure 10:**
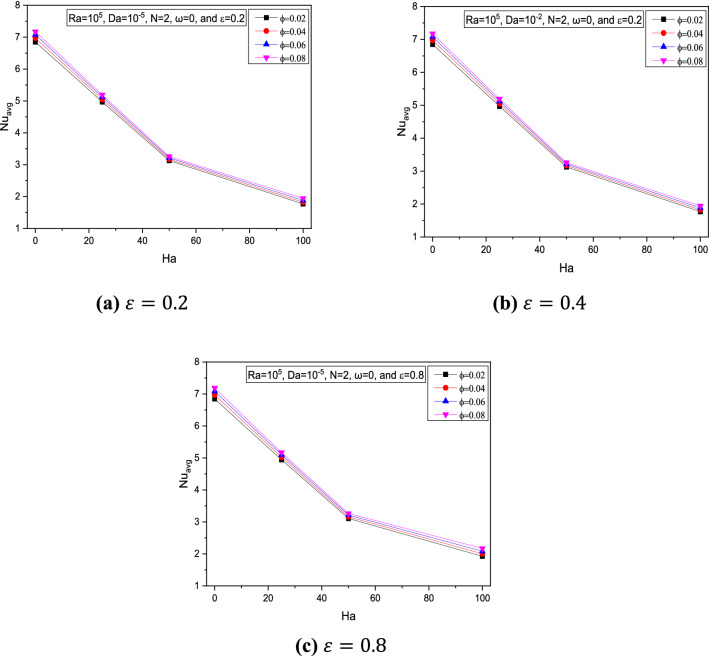
Variations of $Nu_{avg}$ with $Ha$ for various $\phi$ at $Ra = 10^{5}$, $Da = 10^{ - 5}$, $N = 2$ and $\omega = 0$ at (**a**) $\varepsilon = 0.2$, (**b**) $\varepsilon = 0.4,$ and (**c**) $\varepsilon = 0.8$.

It is noticed through Figs. 9 and 10 that for increasing $Ha$, the average Nusselt number $Nu_{avg}$ was reduced due to flow restrictions experienced by the magnetic field intensity.

### Effect of porosity

Compare to normal fluid flow problems, the porosity becomes a vital parameter in nanofluid flows involving porous medium. This may be due to the suspended nanoparticles and their nature. Similar to the effects of Darcy number $Da$, the porosity $\varepsilon$ also possesses clustered streamlines around porous media but in some smaller way. As the increased porosity $\varepsilon$ facilitates the flow across the cavity, the streamlines are altered limitedly.

**Figure 11:**
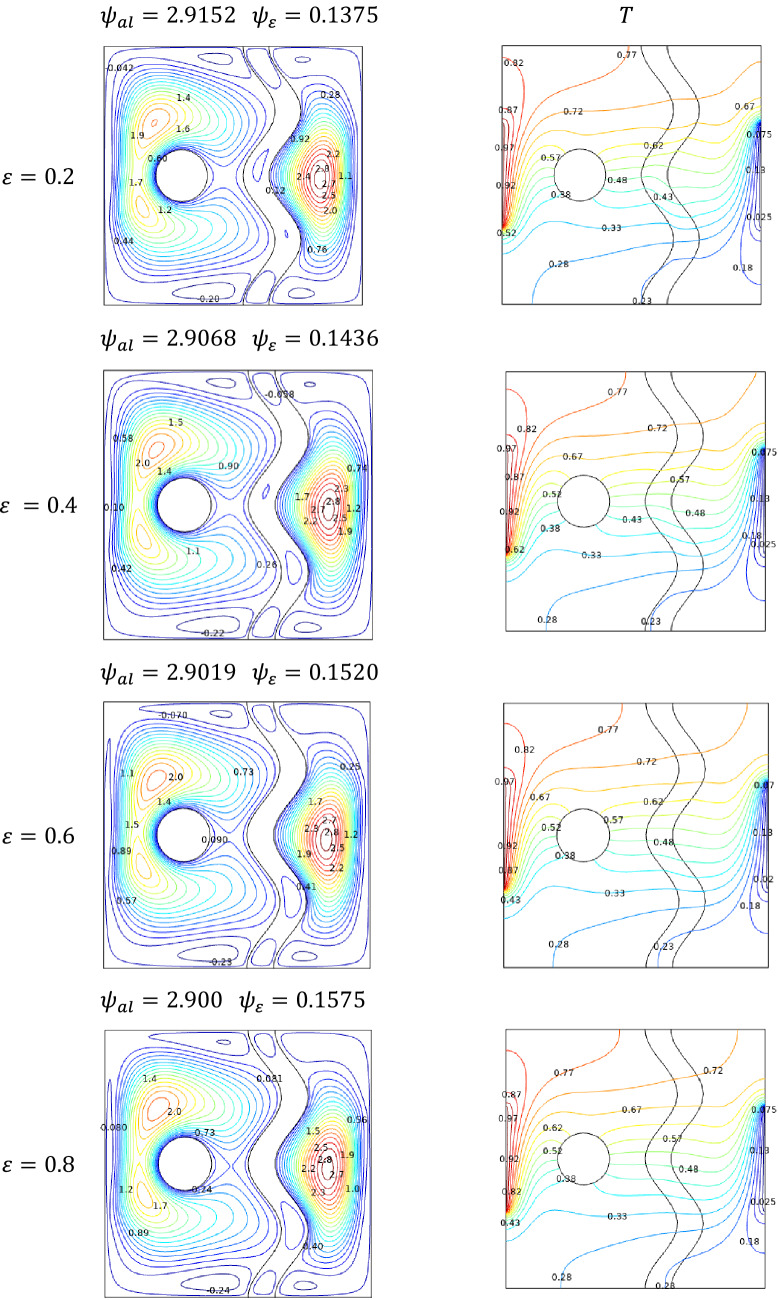
Variations of the streamlines (left), isotherms (right) with various porosity values ($\varepsilon$) at, $Ra = 10^{5}$, $Ha = 0$, $N = 2$, $\phi = 0.02$, $Da = 0.01$, and $\omega = 0.$

Regarding isotherms in Fig. 11, the effect of porosity seems to be minimal. This was observed through the graphs that no such significant alterations happened.

### Effect of solid volume fraction

**Figure 12:**
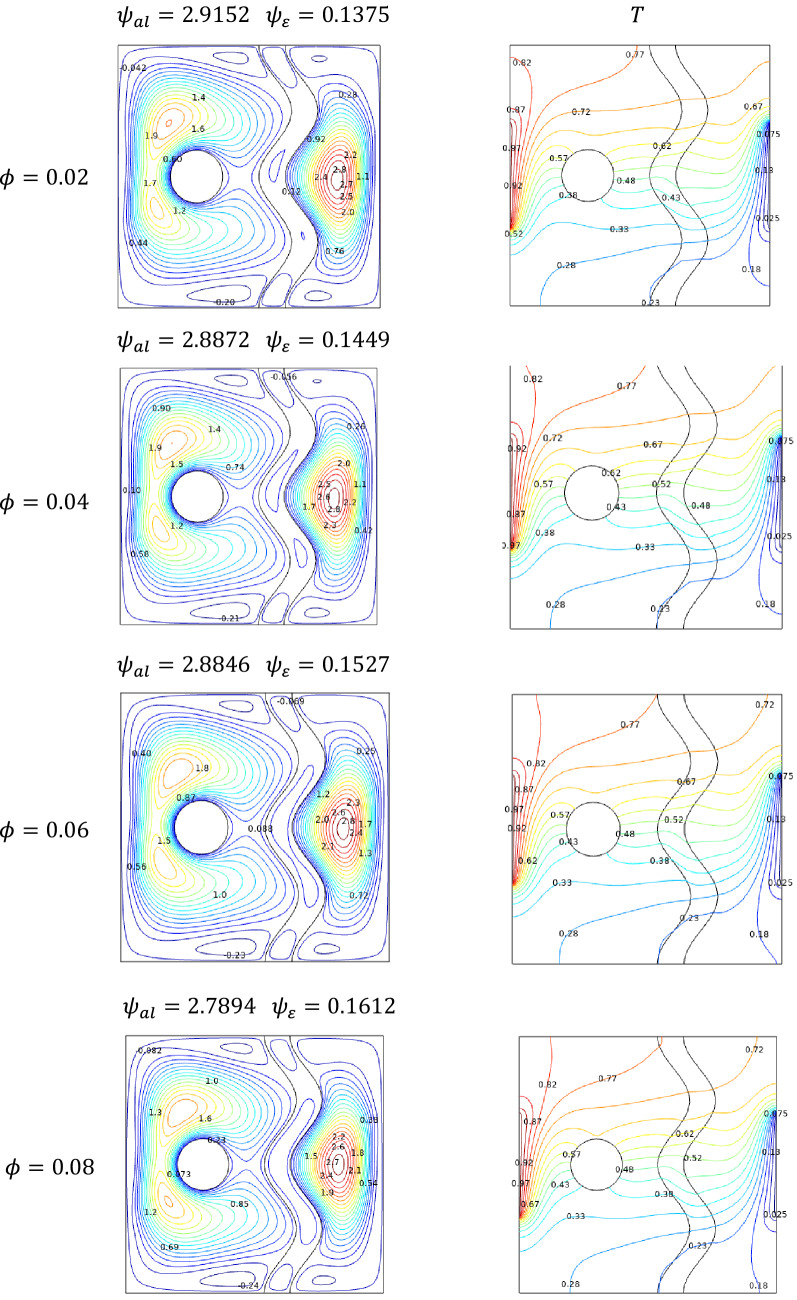
Variations of the streamlines (left), (right) with nanoparticles concentrations ($$\phi$$) at, $$Ra = 10^{5}$$, $$Ha = 0$$, $$N = 2$$, $$\varepsilon = 0.2$$,$$ Da = 0.01$$ and $$\omega = 0.$$

The concentration of solid volume fraction reflects in the quality of nanofluid in terms of its fluidity and thermal efficiency is considered. The influence of solid volume fraction over the streamlines can be noted in either side of the rotating cylinder and behind the undulated region. As the volume fraction gets increased the streamline contours seems to be getting faded due to slowness developed in the cavity by the added particle fraction. It also reflects in the isotherms plots that slower flow grasp more heat from the hotter side which can be seen in Fig. 12.

### Effect of rotating speed

**Figure 13:**
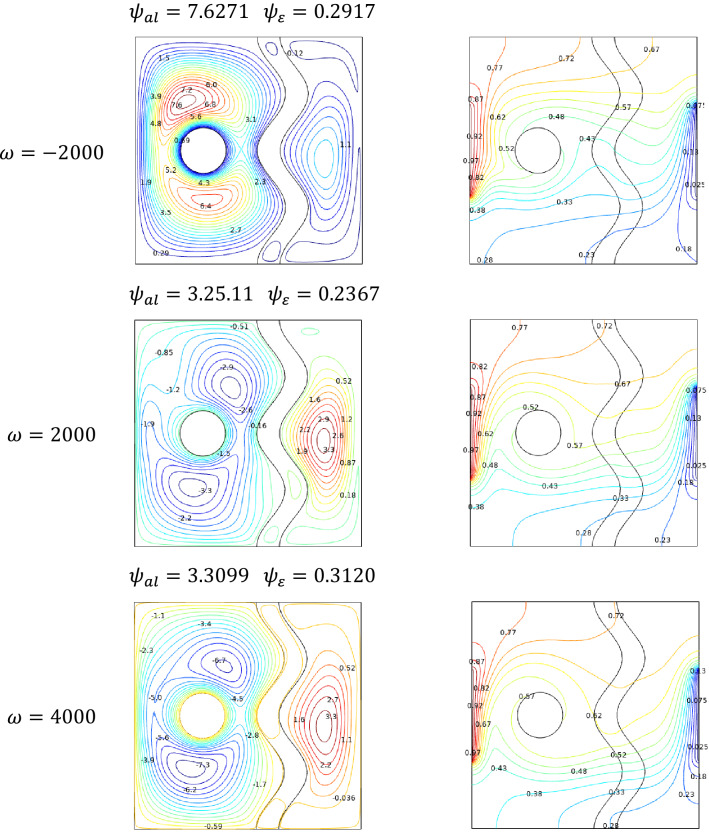
Variations of the streamlines (left), isotherms (right) with various rotational speeds ($\omega$) at, $Ra = 10^{5}$, $Ha = 0$, $N = 2$, $\phi = 0.02$, $\varepsilon = 0.4$ and $Da = 0.01$.

The rotation speed study has been carried out for two cases such as positive and negative values of Rotation speed $\omega$. The findings in Fig. 13 show that for negative rotation speed, the fluid from the hotter wall side rotates over the cavity, which seems to be the maximum coverage of the cavity with hotter fluid. As the rotation speed tends towards positive, the cooler streams spread across the cavity. The isotherms also support the claim considered above. Hotter isotherms tend to rotates from the clockwise direction, while the cooler isotherms tend to rotate in an anti-clockwise direction for higher values of Rotation speed $\omega$.

### Effect of undulation

**Figure 14:**
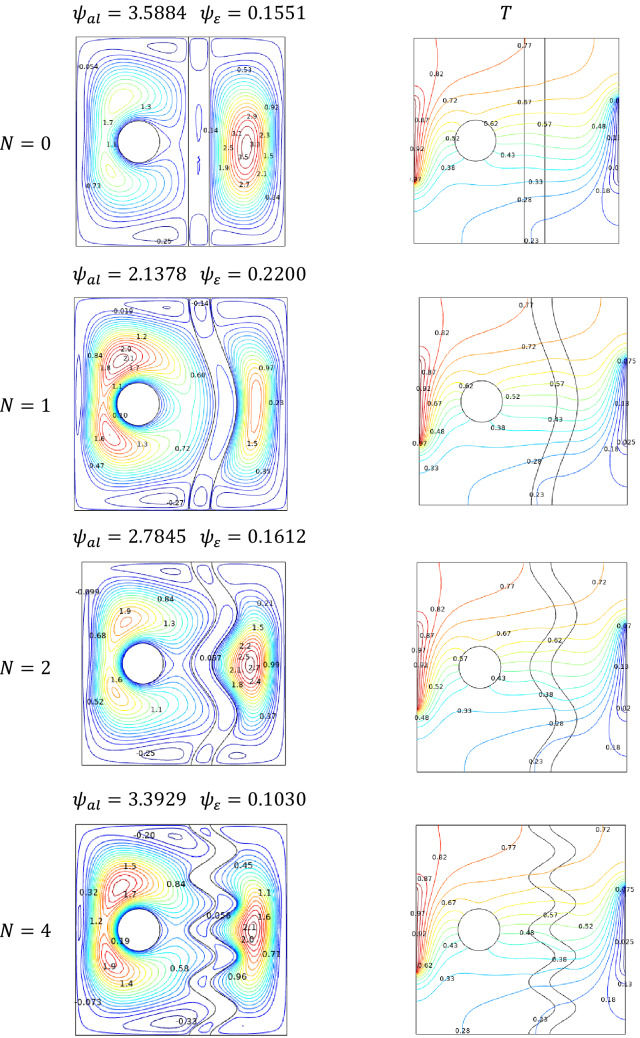
Variations of the streamlines (left), isotherms (right), with undulation $N$ at, $Ra = 10^{5}$, $Ha = 0$, $\varepsilon = 0.4$, $Da = 0.01$, $\phi = 0.08$ and $\omega = 0.$

Variation in undulation values increases the flow fluctuations across the cavity. It is clear through the streamlines and isotherms for both decreased ($N = 1$) and increased ($N = 4$) values of undulations. It is shown in Fig. 14, that the formation of contour in the cavity gets increased. Especially around the porous structure and thermally varied sidewalls.

**Figure 15:**
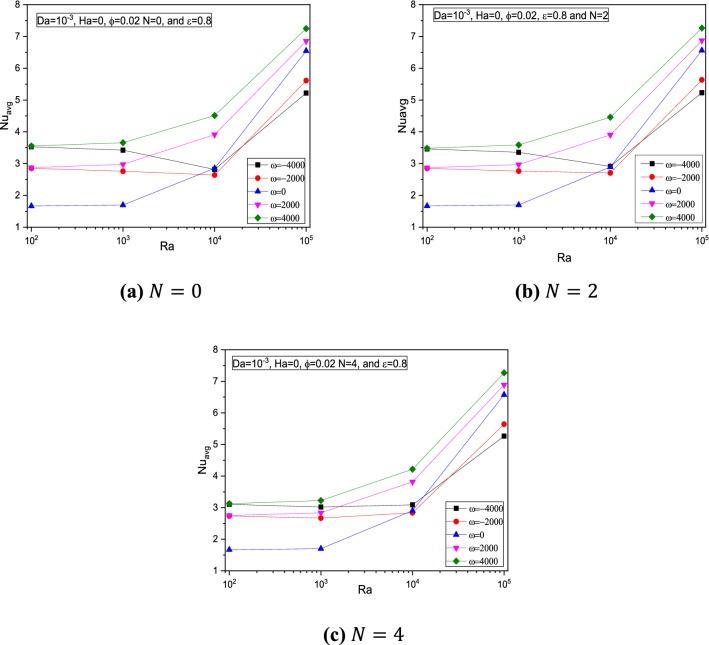
Variations of $Nu_{avg}$ with $Ra$ for various $\omega$ for $Da = 10^{ - 3}$, $Ha = 0$,$\varepsilon = 0.8$, and $\phi = 0.02$ at (**a**) $N = 0$, (**b**) $N = 2$, and (**c**) $N = 4$.

**Figure 16:**
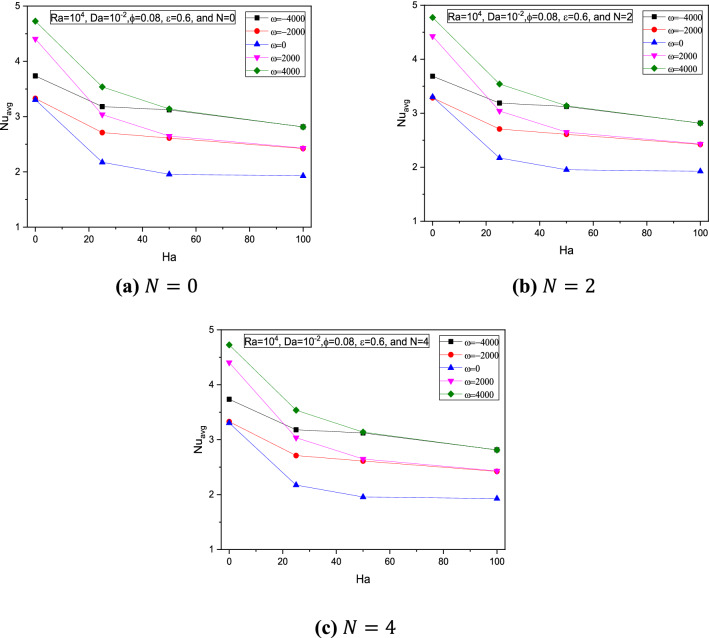
Variations of $Nu_{avg}$ with $Ha$ for various $\omega$ at $Ra = 10^{4}$, $Da = 10^{ - 2}$,$\varepsilon = 0.6$, and $\phi = 0.08$ at (**a**) $N = 0$, (**b**) $N = 2$, and (**c**) $N = 4$.

Exceptionally for Rotation speed $\omega$, it is observed that the average Nusselt number $Nu_{avg}$ gets reduces from the initial state around the values of $Ra = 10^{4}$, and then it hikes continuously as it is shown in Fig. 15. It is noticed through Fig. 16 the average Nusselt number $Nu_{avg}$ got reduced due to flow restrictions experienced by the magnetic field intensity at any rotational speed.

### Entropy generation

**Figure 17:**
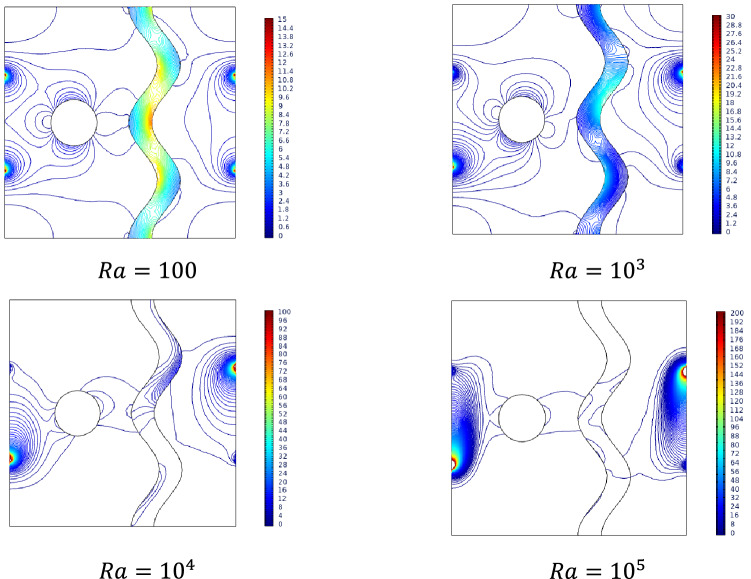
Variations of the general entropy with various Rayleigh number $Ra$ at $Ha = 0$, $N = 2$, $Da = 0.01$, $\phi = 0.02$, $\varepsilon = 0.2$, and $\omega = 0$.

Through Fig. 17, it can be seen that the entropy generation $S_{gen}$ seems to be triggered from both the thermal ends of the cavity. Pair of contours formed in both ends along with the increased entropy changes in porous region for lower Rayleigh number $Ra$. This state gets reversed for increasing values of $Ra$. The dual contours become single in both ends, the heat end contour drags down while the other claims up. The porous part of the cavity gets away with the entropy variation for a higher value of $Ra$.

**Figure 18:**
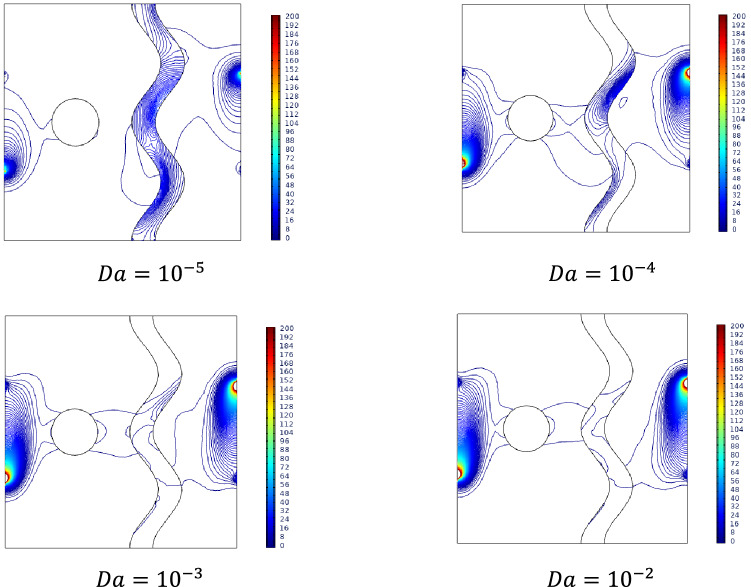
Variations of the general entropy with various Darcy number $Da$ at $Ra = 10^{5}$, $Ha = 0$, $N = 2$, $\phi = 0.02$, $\varepsilon = 0.2$ and $\omega = 0.$

Figure 18 indicates that for the lower Darcy number $Da$, the fluid struggles to enter into the porous medium caused accumulation around it. The graph of entropy generation $S_{gen}$ reflects the state of entropy loss in fluid due to its permeability restrictions through porous media. For higher values of $Da$, the flow gets into the other side to have only entropy losses in two spots of heat variations.

**Figure 19:**
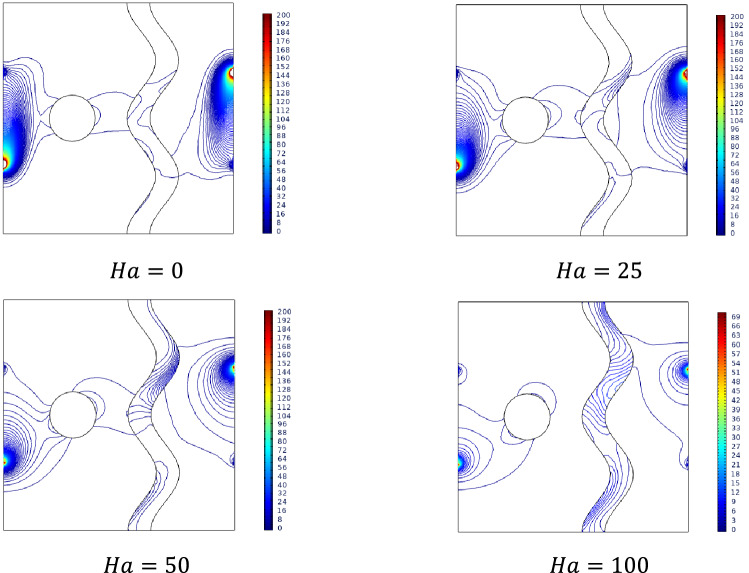
Variations of the general entropy with various Hartmann number $Ha$ at $Ra = 10^{5}$, $Da = 0.01$, $N = 2$, $\phi = 0.02$, $\varepsilon = 0.2$ and $\omega = 0.$

Entropy generation acts opposite to that for the Rayleigh number variations compared to the Hartmann number $Ha$. As it is clearly shown in Fig. 19, the contours start to build from the two rear sides and as the magnetic influence increases, they simultaneously move towards the middle part of the cavity and splits into two contours on each side.

**Figure 20:**
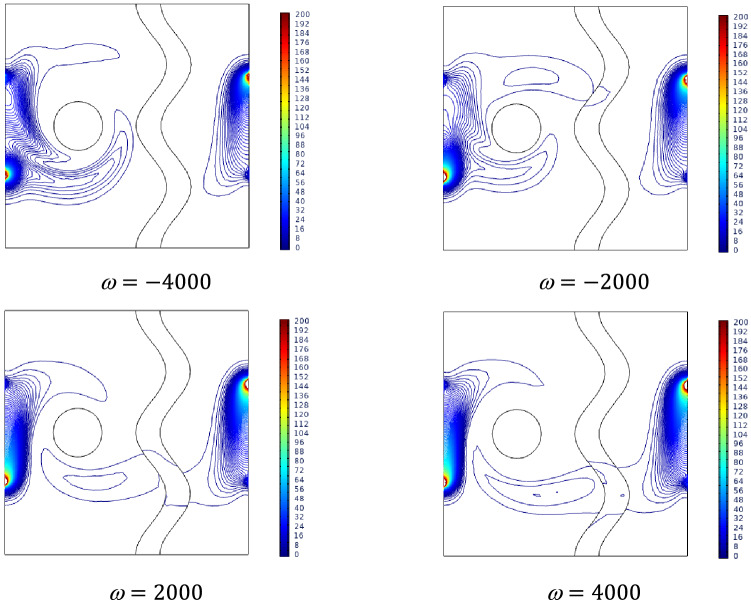
Variations of the general entropy with various rotational speeds $\omega$ at $Ra = 10^{5}$, $Ha = 0$, $N = 2$, $\phi = 0.02$,$\varepsilon = 0.4$ and $Da = 0.01$.

Respective to the rotation direction of the fluid inside the cavity, Fig. 20 illustrates that the entropy generation $S_{gen}$ also shifted clockwise to anticlockwise for increasing values of Rotation speed $\omega$.

**Figure 21:**
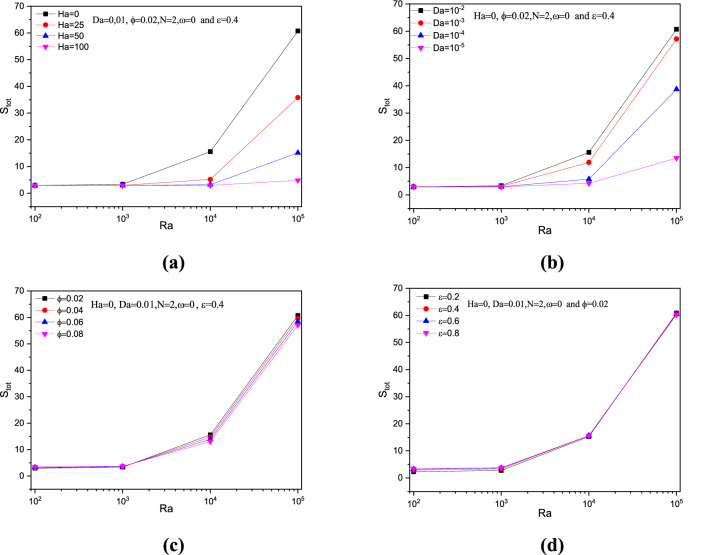
Variations of total entropy with various Rayleigh number $Ra$ for variation $Ra$, $Ha$,$\phi$, $\varepsilon$ at $N = 2$ and $\omega = 0$.

Similar to the average Nusselt number, the entropy generation $S_{gen}$ increased for Rayleigh number greater than $10^{2}$. Entropy control for this problem under Rayleigh number manipulation can be done using parameters like Hartmann number $Ha$ and solid volume fraction $\phi$ while the other parameters can assist entropy as it is shown in Fig. 21.

**Figure 22:**
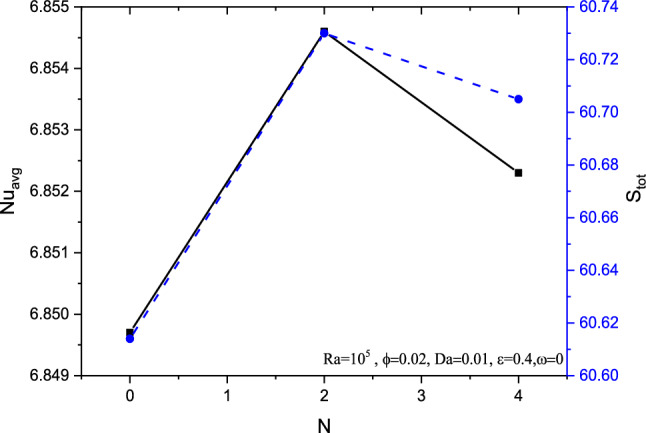
Variations of $Nu_{avg}$ and total entropy with various $N$, for $Ra = 10^{5}$, $Ha = 0$, $\omega = 0$, $Da = 0.01$ and $\varepsilon = 0.4$.

Regarding undulation parameter $N$ impact in Fig. 22, both the average Nusselt number $Nu_{avg}$ and the entropy generation $S_{gen}$ gets increased to the value of undulation $N = 2$. Later both tend to get reduced, especially the Nusselt number drops more than that of entropy generation. This reflects the fact that, more undulations makes the flow and its nanoparticle suspension more difficult which reduces the heat transfer process and simultaneously the entropy generation and total entropy across the system.

## Conclusions

Through the parametric study based on finite element method towards the Ag/Al_2_O_3_-water hybrid nanofluid which filled inside the porous layered enclosure influenced by magnetic field over the rotating cylinder, the following conclusion is made:

- The paired streamline nearer to the heated wall and single cooled were induced for lower values of Rayleigh number, Darcy number higher values of Hartmann number, increased rotating speed and higher values of undulation.
- Except for the rotation speed parameter, heated isotherms claim the top of the cavity and cooled isotherms claim the bottom of it for varied values of physical parameters.
- Negative values of the rotation speed parameter cause the isotherms to revolve in a clockwise direction, whilst positive values cause the isotherms to rotate in an anticlockwise manner.
- Higher values of Rayleigh number and porosity, also lower Hartmann number, lower Darcy number, and lower solid volume fraction characterized higher entropy generation throughout the porous medium.
- Increased Rayleigh number, positive rotation speed, solid volume fraction, and rising Darcy number improved the heat transfer rate across the cavity in the contrary of higher Hartmann number.
- Both the streamlines and isotherms are decreased at $N = 1$ and increased at $N = 4$ values of undulations. Entropy generation $S_{gen}$ gets higher at the undulation of $N = 2$.

## Data Availability

The results of this study are available only within the paper to support the data.

## List of symbols

B_0_: Intensity of magnetic field
$d_{m}$: Diameter of cylinder (${\text{m}}$)
$$C_{p}$$: Heat capacitance $$\left( {{\text{J/kg}}\,{\text{K}}} \right)$$
$$Da$$: Darcy number
$$F_{c}$$: Forchheimer coefficient
$$g$$: Gravitational acceleration ($${\text{m/s}}^{2}$$)
$$Ha$$: Hartmann number
$$k$$: Thermal conductivity $$\left( {{\text{W/m}}\,{\text{k}}} \right)$$
$$K$$: Permeability
$L,H$: Long and height of the cavity (${\text{m}}$)
$$N$$: Undulations number
$$Nu$$: Nusselt number
$Nu_{avg}$: Average Nusselt number
$Nu_{loc}$: Local Nusselt number
$$p$$: Pressure ($${\text{N}}/{\text{m}}^{2}$$)
$$P$$: Dimensionless pressure
$$Pr$$: Prandtl number
$$Ra$$: Rayleigh number
$S_{gen}$: Dimensionless entropy generation
$$T$$: Temperature ($${\text{K}}$$)
$T_{h}$: Hot part temperature (${\text{K}}$)
$T_{c}$: Cold part temperature (${\text{K}}$)
$$u,v$$: Velocity components ($${\text{m/s}}$$)
$U,V$: Nondimensional velocity components
$$x,y$$: Coordinate ($${\text{m}}$$)
$$X,Y$$: Dimensionless coordinate

## Greeks symbols

$$\alpha$$: Thermal diffusivity ($${\text{m}}^{2} /{\text{s}}$$)
$\beta$: Thermal expansion coefficient ($1/{\text{K}}$)
$$\varepsilon$$: Porosity
$$\theta$$: Dimensionless temperature
$$\phi$$: Volume fraction
$$\mu$$: Dynamic viscosity ($${\text{kg/m}}\,{\text{s}}$$)
$$\nu$$: Kinematic viscosity ($${\text{m}}^{2} /{\text{s}}$$)
$$\rho$$: Density ($${\text{kg}}/{\text{m}}^{3}$$)
$$\sigma$$: Electrical conductivity $$\left( {{\Omega }\,{\text{m}}} \right)^{ - 1}$$
$\psi$: Non-dimensional stream function
$$\omega$$: Rotational speed $$\left( {\text{m/s}} \right)$$

## Subscripts

$$avg$$: Average
$$bf$$: Base fluid
$$c$$: Cold
$$h$$: Hot
$$hnf$$: Hybrid-nanofluid
$$loc$$: Local
$$np$$: Nanoparticles
